# Supplementary material for: Genome-Wide Pleiotropy Study Identifies Association of PDGFB with Age-Related Macular Degeneration and COVID-19 Infection Outcomes
Source: J Clin Med. 2022 Dec 23;12(1):109. doi: 10.3390/jcm12010109 (PMC9821609; doi:10.3390/jcm12010109)
Supplement: Supplementary file 1 [file jcm-12-00109-s001.zip › jcm-2066460-supplementary.pdf]

Table S1. Overview of the publicly available summary-level data of genetic associations used for the analysis.

Phenotype	Description	Sample Size	Cases	Controls	Population	PMID
AMD	AMD cases vs controls	33,976	16,144	17,832	EUR	26691988
COVID-19	Critical Covid vs population	1,388,342	5,101	1,383,241	EUR	34237774
	Hospitalized Covid vs population	1,887,658	9,986	1,877,672		
	Covid infection vs population	1,683,768	38,984	1,644,784		

PMID: PubMed accession ID.

Table S2. Sample demography of gene expression dataset derived from 96 peripheral blood samples obtained from 32 COVID-19 patients and 8 controls at multiple time points, corresponding to different disease stages, measured from symptom onset (early: 19 samples within 10 days; middle: 36 samples between 11 and 20 days; late: 22 samples >21 days or later). This COVID-19 expression data was obtained from Gene Expression Omnibus (GEO; accession ID: GSE161731).

Age group	Control	Early	Middle	Late
**N (Females)**	19 (8)	19 (6)	36 (15)	22 (11)
**Age Mean (SD)**	18.37 (0.6)	46.44 (19.7)	41.06 (16.4)	41.82 (13.4)
**10-20**	19	2	1	1
**20-30**	0	4	13	7
**30-40**	0	5	8	2
**40-50**	0	0	3	5
**50-80**	0	8	11	7

Table S3. Sample demography of AMD gene expression dataset derived from human retinal pigment epithelium (RPE)/choroid tissues obtained from 9 AMD cases and 6 age-matched healthy controls, that were downloaded from the Gene Expression Omnibus (GEO; accession ID: GSE50195).

	**Total**	**AMD**	**Controls**
**N (Females)**	16 (8)	9 (5)	6 (3)
**Age Mean (SD)**	83.69 (6.01)	84.22 (6.55)	83.00 (5.66)

Table S4. Overview of the genetic variants used as instrumental variables for testing causal inference between AMD and COVID-19 traits (critical illness, hospitalization, and infection). We selected 23 uncorrelated (clumped at correlation threshold $r^2 < 0.01$) and common (MAF > 5%) genetic variants as instrumental variables that were associated with AMD at a p-value level of 5×10^{-8} or smaller. The table additionally includes summary-level data (beta coefficients of genetic association, their standard error and corresponding p-value) for AMD as exposure and three COVID-19 phenotypes, including critical illness, hospitalization, and infections, as outcomes. CH: chromosome; EA: effect allele; EAF: effect allele frequency; β: effect estimate; SE: standard error.

				**AMD**			**COVID-19 Critical Illness**			**COVID-19 Hospitalization**			**COVID-19 Infection**		
**SNP**	**CH**	**EA**	**EAF**	**β**	**SE**	**P**	**β**	**SE**	**P**	**β**	**SE**	**P**	**β**	**SE**	**P**
rs10801558	1	G	0.33	-0.96	0.02	2.5×10^{-308}	-0.01	0.03	0.58	-0.02	0.02	0.4	-0.02	0.01	0.09
rs62247658	3	T	0.45	-0.13	0.02	1.8×10^{-14}	-0.01	0.03	0.72	0.02	0.02	0.38	0.00	0.01	0.99
rs56339461	3	G	0.16	-0.13	0.02	2.6×10^{-9}	0.01	0.05	0.86	0.01	0.03	0.68	0.01	0.01	0.36
rs10033900	4	C	0.49	-0.14	0.02	5.4×10^{-17}	0.00	0.03	0.86	-0.02	0.02	0.27	-0.02	0.01	0.08
rs429608	6	A	0.12	-0.56	0.03	1.2×10^{-103}	-0.01	0.04	0.73	0.00	0.03	0.94	0.00	0.01	0.75
rs7803454	7	T	0.2	0.12	0.02	4.8×10^{-9}	0.00	0.03	0.90	0.01	0.02	0.57	0.02	0.01	0.21
rs1142	7	T	0.36	0.10	0.02	1.4×10^{-9}	0.04	0.03	0.14	-0.02	0.02	0.28	-0.01	0.01	0.58
rs10781182	9	G	0.32	-0.11	0.02	2.6×10^{-9}	-0.01	0.04	0.77	0.02	0.02	0.32	0.00	0.01	0.67
rs401186	9	T	0.2	-0.13	0.02	4.1×10^{-10}	0.07	0.04	0.08	0.05	0.03	0.07	0.01	0.01	0.37
rs11200633	10	T	0.32	1.04	0.02	2.5×10^{-308}	-0.03	0.03	0.43	-0.02	0.02	0.28	0.00	0.01	0.96
rs11624933	14	G	0.14	0.16	0.03	2.5×10^{-10}	-0.01	0.06	0.9	0.01	0.03	0.70	0.01	0.01	0.30
rs61985136	14	T	0.37	0.11	0.02	1.6×10^{-10}	0.05	0.03	0.12	0.01	0.02	0.57	0.02	0.01	0.08
rs2414577	15	C	0.37	-0.13	0.02	4.3×10^{-15}	-0.02	0.03	0.54	-0.01	0.02	0.79	-0.01	0.01	0.46
rs1864163	16	A	0.25	-0.17	0.02	6.3×10^{-19}	-0.04	0.03	0.21	-0.01	0.02	0.63	0.00	0.01	0.82
rs72802342	16	A	0.07	-0.23	0.03	5.0×10^{-12}	0.01	0.07	0.84	0.04	0.04	0.25	0.01	0.02	0.45
rs11080055	17	C	0.48	0.09	0.02	1.0×10^{-8}	-0.01	0.03	0.85	0.01	0.02	0.63	0.02	0.01	0.11
rs6565597	17	T	0.39	0.12	0.02	1.5×10^{-11}	-0.05	0.04	0.20	0.00	0.02	0.97	0.00	0.01	0.99
rs9973159	18	T	0.13	-0.13	0.02	9.4×10^{-8}	0.00	0.05	0.98	-0.02	0.03	0.52	0.00	0.01	0.81
rs11569415	19	A	0.24	0.34	0.02	5.4×10^{-67}	0.01	0.05	0.86	0.00	0.03	0.87	0.00	0.01	0.77
rs6073984	20	G	0.13	-0.15	0.02	3.4×10^{-10}	0.03	0.04	0.45	0.02	0.03	0.37	0.01	0.01	0.45
rs117739907	20	T	0.06	-0.28	0.03	3.2×10^{-16}	0.05	0.05	0.33	0.03	0.04	0.35	0.02	0.02	0.35
rs5754227	22	C	0.12	-0.26	0.03	1.1×10^{-24}	-0.01	0.04	0.79	0.00	0.03	0.97	0.00	0.01	0.93
rs8135665	22	T	0.21	0.13	0.02	5.6×10^{-11}	0.04	0.03	0.20	0.04	0.02	0.10	0.00	0.01	0.87

Table S5. Overview of the genetic variants used as instrumental variables for testing causal inference between the human cytokine Pdgfbb level and COVID-19 outcomes. We selected 4 uncorrelated (clumped at correlation threshold $r^2 < 0.01$) and common (MAF > 5%) genetic variants as instrumental variables that were associated with the Pdgfbb levels at a p-value level of 9×10^{-8} or smaller. The table additionally includes summary-level data (beta coefficients of genetic association, their standard error and corresponding p-value) for Pdgfbb as exposure and three COVID-19 phenotypes, including critical illness, hospitalization, and infections, as outcomes. CH: chromosome; EA: effect allele; EAF: effect allele frequency; β: effect estimate; SE: standard error.

				**Pdgfbb**			**Critical Illness**			**Hospitalization**			**Infection**		
**SNP**	**CH**	**EA**	**EAF**	**β**	**SE**	**P**	**β**	**SE**	**P**	**β**	**SE**	**P**	**β**	**SE**	**P**
rs72777070	2	G	0.11	0.11	0.02	$9.0\text{x}10^{-8}$	-0.03	0.04	0.48	-0.01	0.03	0.65	0.02	0.01	0.02
rs13412535	2	G	0.22	-0.34	0.02	$2.5\text{x}10^{-55}$	-0.02	0.03	0.44	0.00	0.02	0.83	-0.01	0.01	0.42
rs2324229	6	C	0.36	-0.09	0.02	$3.5\text{x}10^{-8}$	-0.03	0.03	0.20	-0.03	0.02	0.15	0.00	0.01	0.94
rs4965869	15	C	0.26	-0.18	0.02	$5.7\text{x}10^{-24}$	-0.02	0.04	0.62	-0.01	0.02	0.54	0.00	0.01	0.98

Table S6. Summary statistics of genome-wide significant ($P < 5.0x10^{-8}$) associations in the MTAG results of AMD and the three COVID-19 outcomes (critical illness, hospitalization, and infections).

Enclosed electronic excel file

Table S7. eQTL association summaries of rs130651 and rs482037 with *CACNA1I* in the whole bloods of the GTEx portal database.

Gene	SNP	Effect Allele	T-statistics	P-Value
CACNA1I	rs130651	A	3.8	$1.4x10^{-4}$
	rs4820371	T	2.3	0.02

Table S8. Summary statistics of *cis*-mQTL associations between the two SNPs (rs130651 and rs4820371) and CpGs located upstream of *PDGFB* (chr22:39619364-39640987) in the GoDMC database (http://www.godmc.org.uk).

SNP	EA	CpG ID	CpG BP*	Distance from PDGFB	Effect	SE	N	P
rs130651	A	cg01416388	chr22:39784597	143610	-0.12	0.01	19065	5.18×10^{-28}
		cg05872129	chr22:39784768	143781	-0.15	0.01	19065	9.23×10^{-43}
		cg24399712	chr22:39784795	143808	-0.15	0.01	19065	2.73×10^{-43}
		cg11247378	chr22:39784981	143994	-0.18	0.01	19065	9.74×10^{-64}
rs4820371	T	cg01093212	chr22:39715155	74168	-0.11	0.01	17974	1.54×10^{-19}
		cg17798944	chr22:39715224	74237	-0.18	0.01	16372	1.55×10^{-45}
		cg02038168	chr22:39784480	143493	-0.12	0.01	17974	2.10×10^{-21}
		cg01416388	chr22:39784597	143610	-0.14	0.01	17974	1.91×10^{-29}
		cg05872129	chr22:39784768	143781	-0.16	0.01	17974	3.92×10^{-41}
		cg24399712	chr22:39784795	143808	-0.16	0.01	17974	5.17×10^{-41}
		cg11247378	chr22:39784981	143994	-0.20	0.01	17974	5.99×10^{-60}

EA: effect allele. CHR: chromosome. BP: base pair position. SE: standard error. N: sample size. *The gene and CpG sites are mapped to the human genome reference build 37.

Table S9. Association summaries for differential gene expression analysis of *PDGFB* in the RNS-Seq data of bloods from COVID-19 patients in three different stages measured from the symptom onset (early ≤10 days, middle 11-20 days, late ≥ 21 days). This COVID-19 RNA-Seq data was obtained from Gene Expression Omnibus (GEO; accession ID: GSE161731).

	**Middle vs. Early**			**Middle/Late vs. Early**		
**Age group**	**FC**	**t**	**P**	**FC**	**t**	**P**
All	0.99	0.52	0.61	0.93	0.24	0.81
> 30	1.02	1.02	0.31	0.92	0.49	0.62
> 40	2.17	2.34	0.03	2.04	1.67	0.10
> 50	2.15	2.19	0.04	1.94	1.36	0.19

FC: fold change of *PDGFB* expression level between the two groups

Table S10. The gene ontology (GO) analysis of the MTAG Result from AMD and the COVID-19 infection. This GO Analysis was conducted using the MAGMA software. The gene-set association significance threshold is set to $8.45x10^{-6}$.

	Infection			
**Gene Set**	**N Genes**	**Beta**	**SE**	**P**
High density lipoprotein particle remodeling	16	1.38	0.25	$1.71x10^{-8}$
Reverse cholesterol transport	16	1.23	0.26	$1.53x10^{-6}$
Humoral immune response mediated by circulating immunoglobulin	44	0.71	0.16	$4.31x10^{-6}$
Extracellular structure organization	396	0.20	0.05	$3.53x10^{-6}$

Table S11. Mendelian randomization associations between AMD and Pdgf-bb as exposures and the COVID-19 (critical illness, hospitalization, and infection) as outcomes using pleiotropy-robust Mendelian randomization approaches including weighted median (WM) and MR-Egger. The intercept of the MR-Egger method was used to test for directional pleiotropy. The estimates are odd ratios for AMD and AMD (complement) and beta for Pdgf-bb.

Exposure	Outcome	MR Method	Estimates	95% CI	P-value
AMD	Critical Illness	WM	1.00	(0.95 - 1.05)	0.97
		MR-Egger (intercept)	0.98	(0.93 - 1.03)	0.40 (0.35)
	Hospitalizat ion	WM	1.00	(0.97 - 1.03)	0.98
		MR-Egger (intercept)	1.00	(0.95 - 1.05)	0.90 (0.88)
	Infection	WM	1.00	(0.98 - 1.02)	1.00
		MR-Egger (intercept)	1.00	(0.99 - 1.02)	0.65 (0.70)
AMD (Complement)	Critical Illness	WM	1.01	(0.96 - 1.07)	0.69
		MR-Egger (intercept)	1.02	(0.97 - 1.07)	0.44 (0.76)
	Hospitalizat ion	WM	1.02	(0.98 - 1.06)	0.38
		MR-Egger (intercept)	1.01	(0.98 - 1.03)	0.64 (0.14)
	Infection	WM	1.01	(0.99 - 1.03)	0.16
		MR-Egger (intercept)	1.00	(0.97 - 1.02)	0.84 (0.052)
Pdgf-bb	Critical Illness	WM	0.07	(-0.10 - 0.23)	0.42
		MR-Egger (intercept)	0.03	(-0.14 - 0.20)	0.72 (0.76)
	Hospitalizat ion	WM	0.01	(-0.09 - 0.12)	0.80
		MR-Egger (intercept)	-0.08	(-0.17 - 0.01)	0.08 (0.16)
	Infection	WM	0.02	(-0.03 - 0.08)	0.37
		MR-Egger (intercept)	0.01	(-0.06 - 0.07)	0.85 (0.54)

CI: Confidence interval. WM: Weighted median.

Table S12. Association summaries of the human cytokine Pdgfbb levels between COVID-19 cases and controls. The positive effect direction means that the mean levels of the cytokine Pdgfbb were higher in the cases compared to the controls.

Study	PMID	COVID-19 Cases (#)	COVID-19 free Controls	Effect direction	P-value
Huang et al. 2020	31986264	ICU-Care COVID-19 cases (13)	28	+	0.013
Huang et al. 2020	31986264	No ICU COVID-19 cases (28)	28	+	0.009
Petrey et al. 2021*	32930456	COVID-19 cases (22)	22	+	<0.001

**Petrey et al.* analyzed the cytokine Pdgf-bb together with Pdgf-ab. PMID: PubMed accession ID.

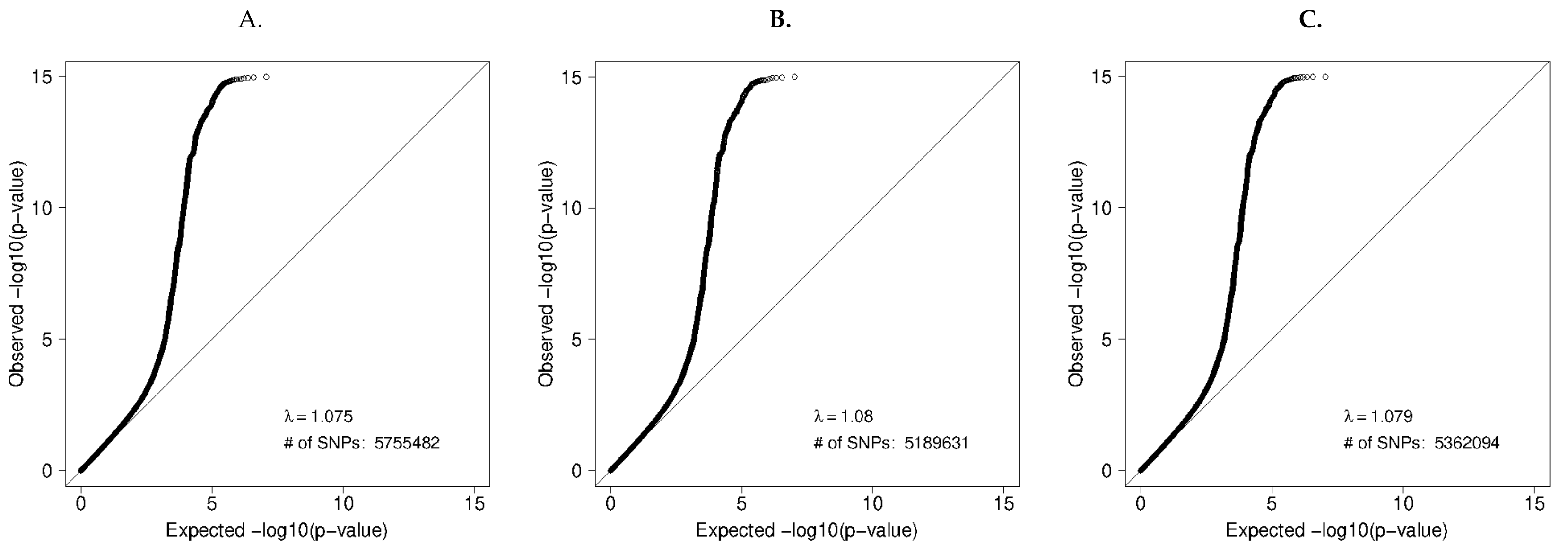

Figure S1. QQ plots of the MTAG results for AMD with the three COVID-19 outcomes: **A.** critical illness, **B.** hospitalization, and **C.** infections.

A.

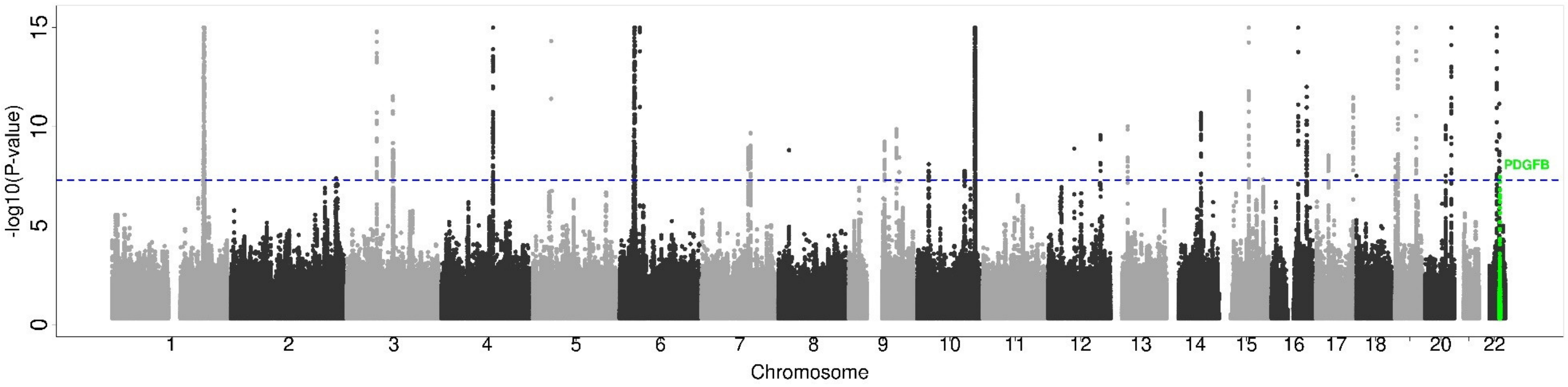

-log10(P-value)
0
5
10
15
PDGFB
1
2
3
4
5
6
7
8
9
10
11
12
13
14
15
16
17
18
20
22
Chromosome

B.

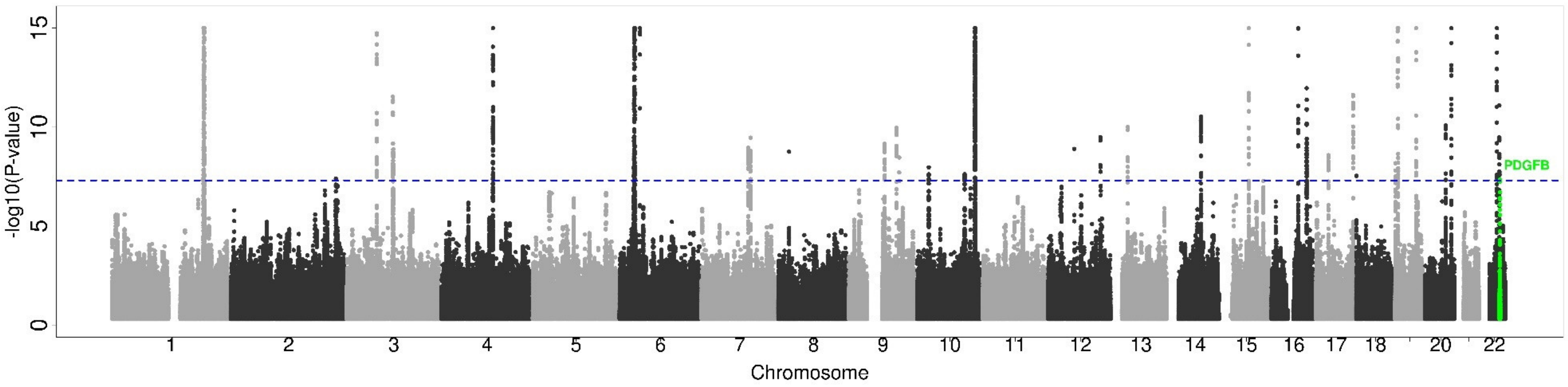

-log10(P-value)
0
5
10
15
PDGFB
1
2
3
4
5
6
7
8
9
10
11
12
13
14
15
16
17
18
20
22
Chromosome

C.

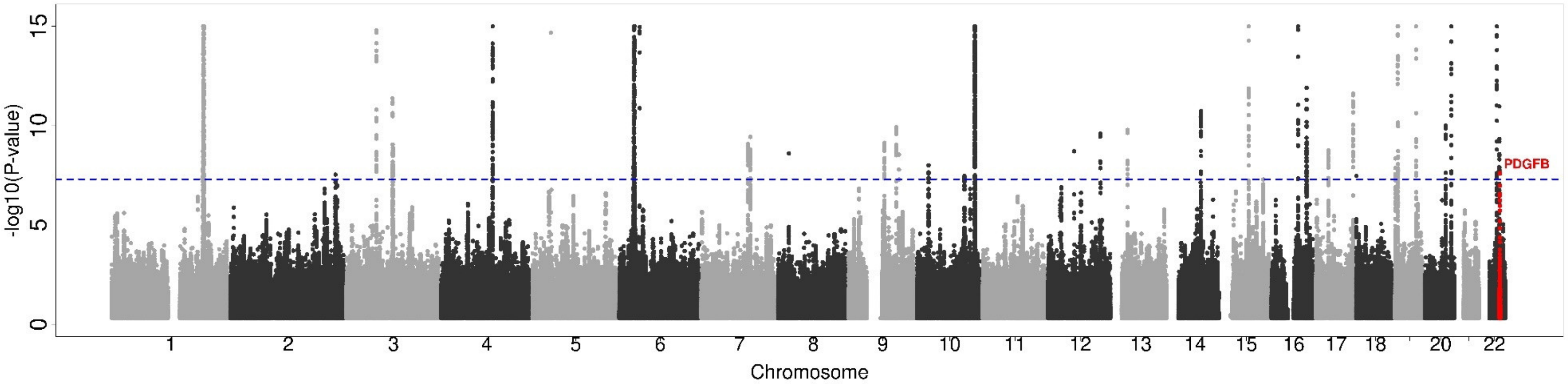

Figure S2. Manhattan plots of the MTAG results for AMD with the three COVID-19 outcomes: **A.** critical illness, **B.** hospitalization, and **C.** infections.

A. critical illness, **B.** hospitalization, and **C.** infections

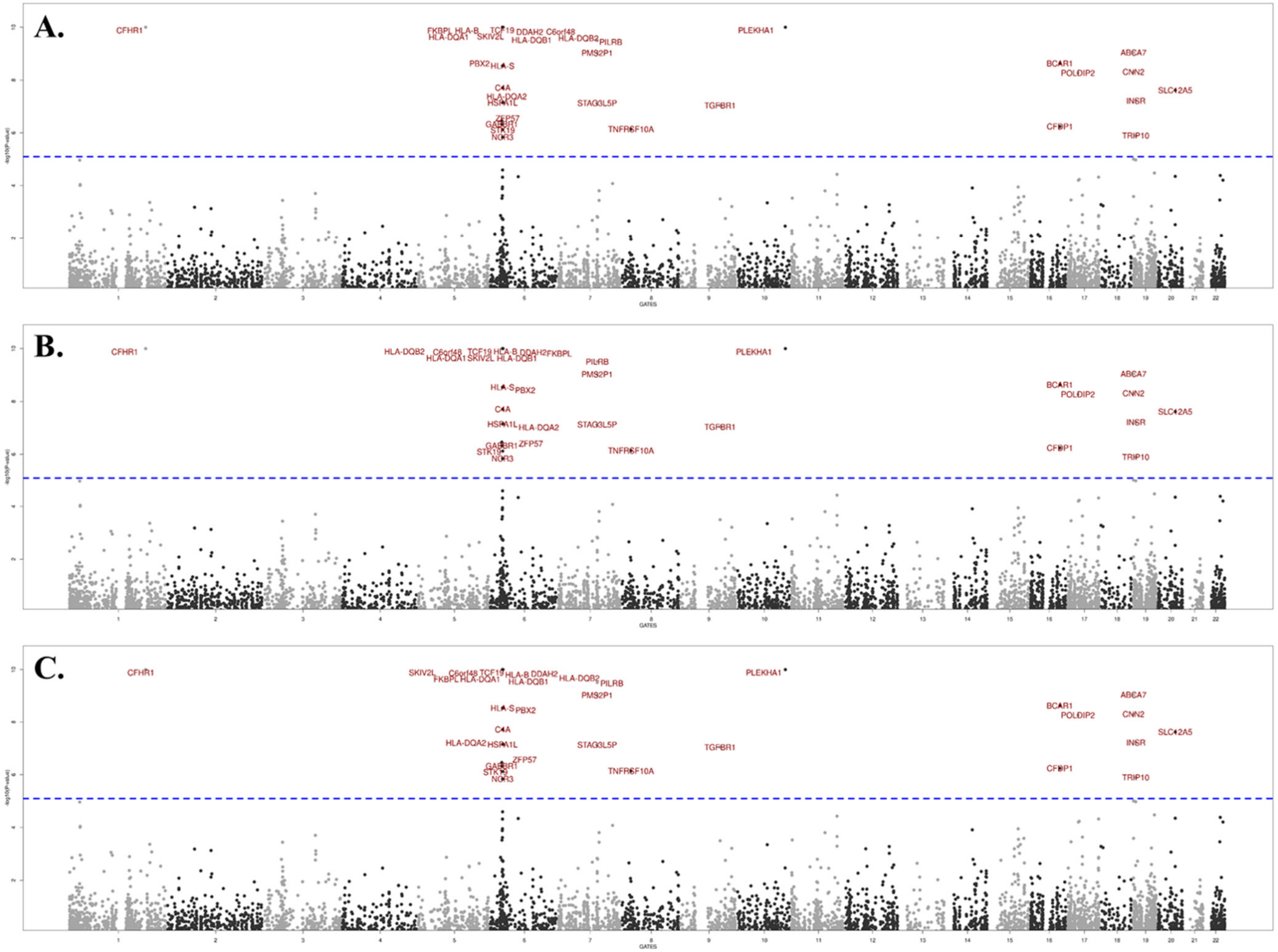

Figure S3. Manhattan plots of S-Predixcan results in the blood tissue using the MTAG results for AMD with the three COVID-19 outcomes including.

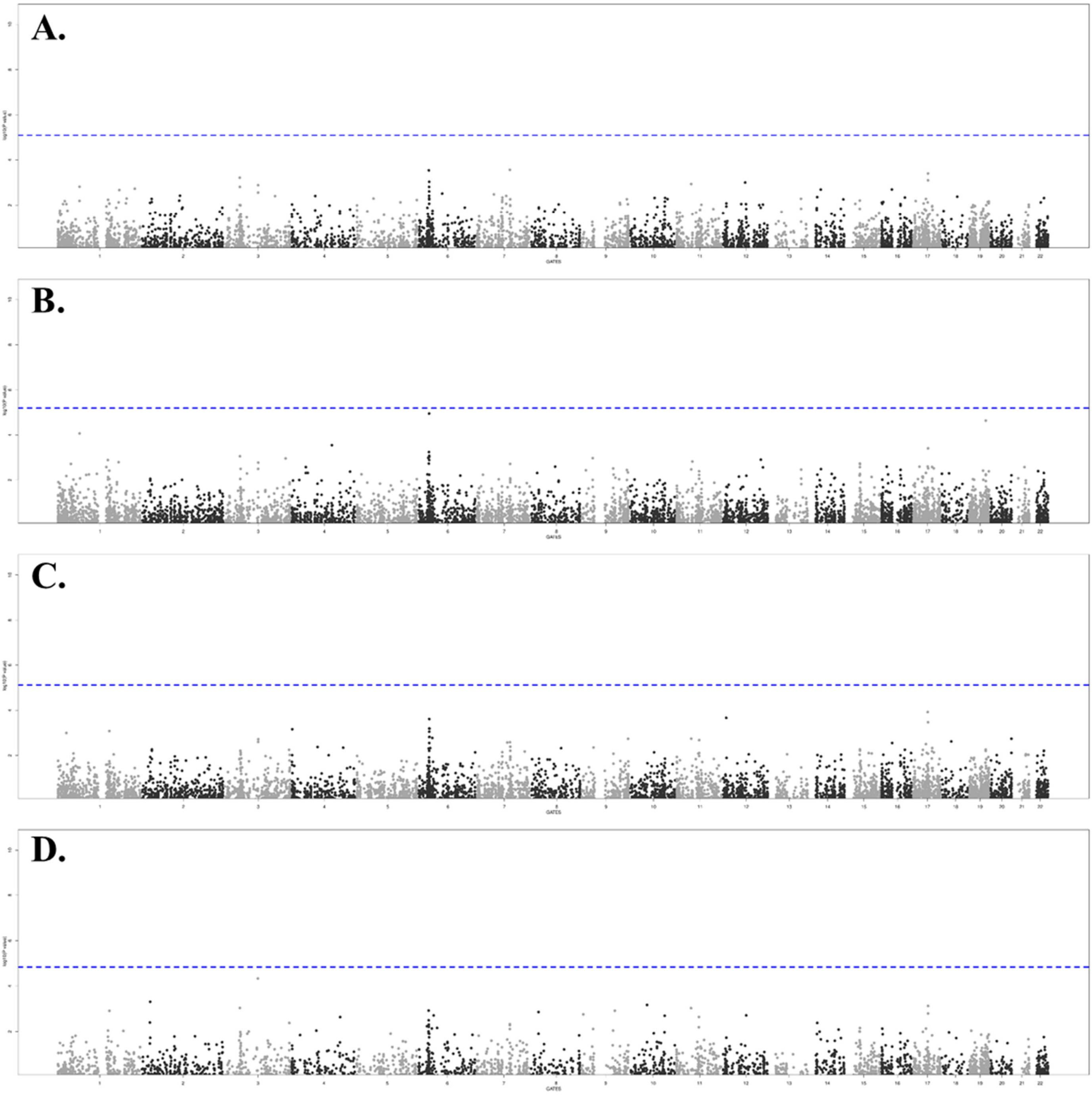

Figure S4. Manhattan plots of S-Predixcan results in **A.** bloods, **B.** lung, **C.** aorta (artery) and **D.** coronary (artery) using the MTAG results for AMD with the COVID-19 critical illness.

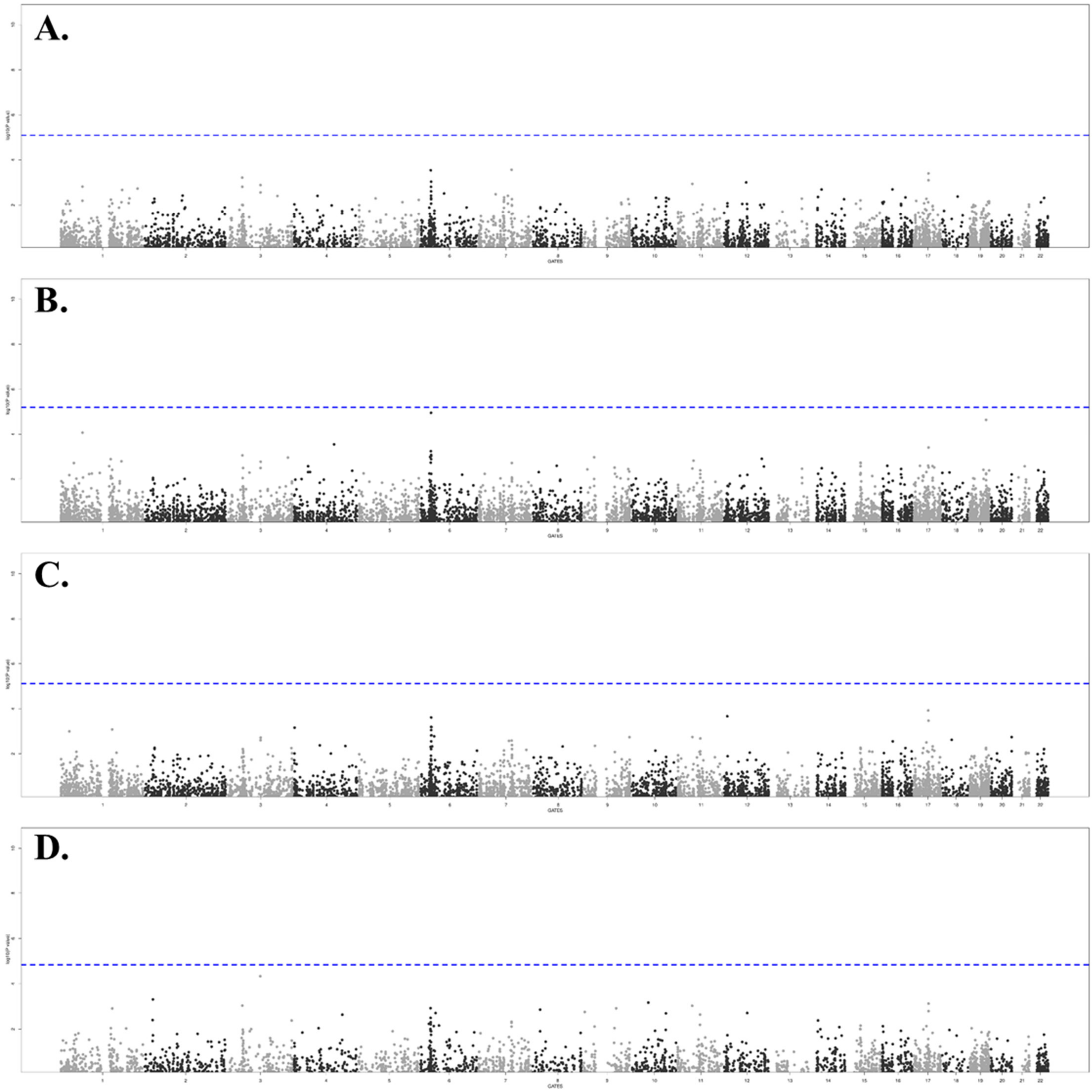

Figure S5. Manhattan plots of S-Predixcan results in **A.** bloods, **B.** lung, **C.** aorta (artery) and **D.** coronary (artery) using the MTAG results for AMD with the COVID-19 hospitalization.

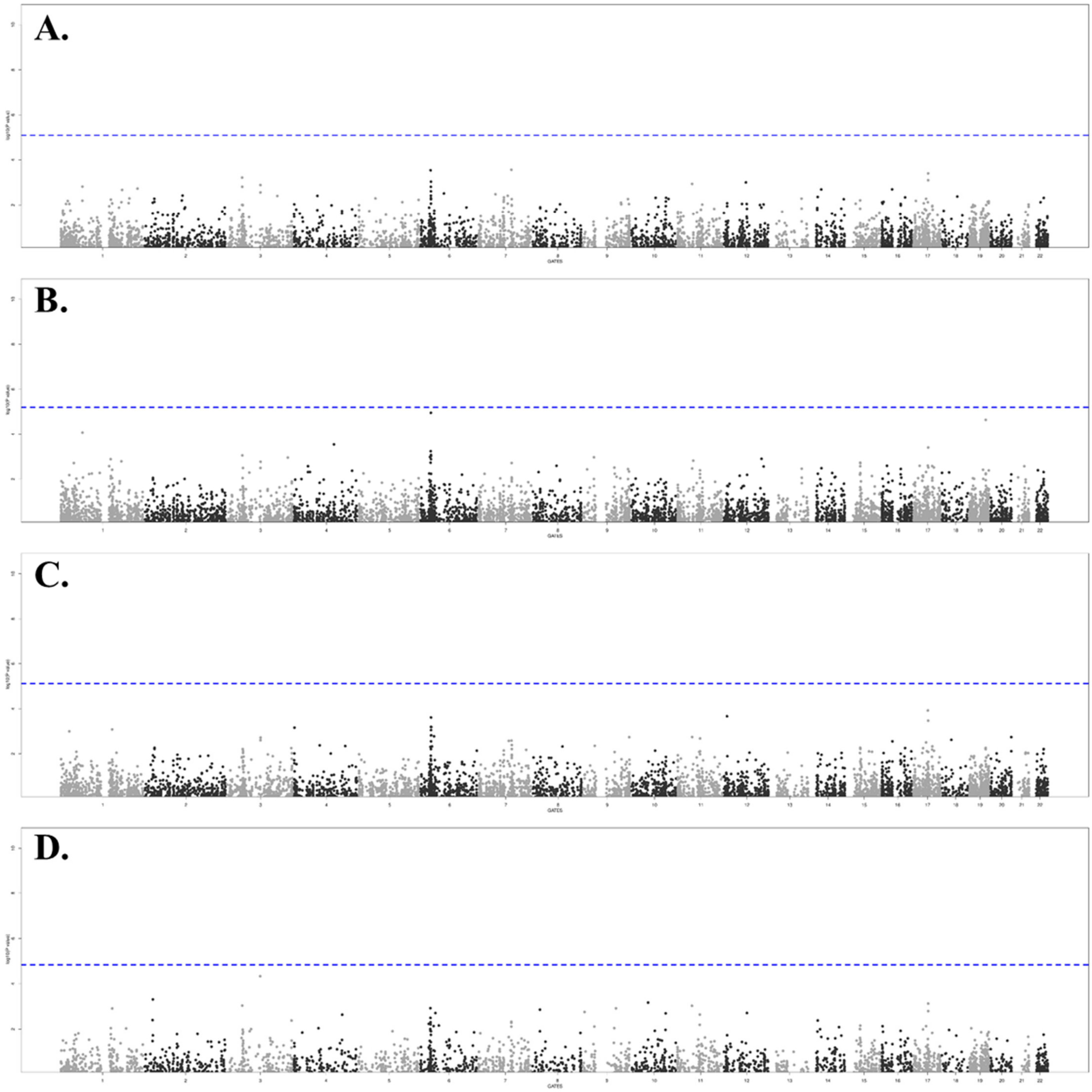

Figure S6. Manhattan plots of S-Predixcan results in **A.** bloods, **B.** lung, **C.** aorta (artery) and **D.** coronary (artery) using the MTAG results for AMD with the COVID-19 infection.

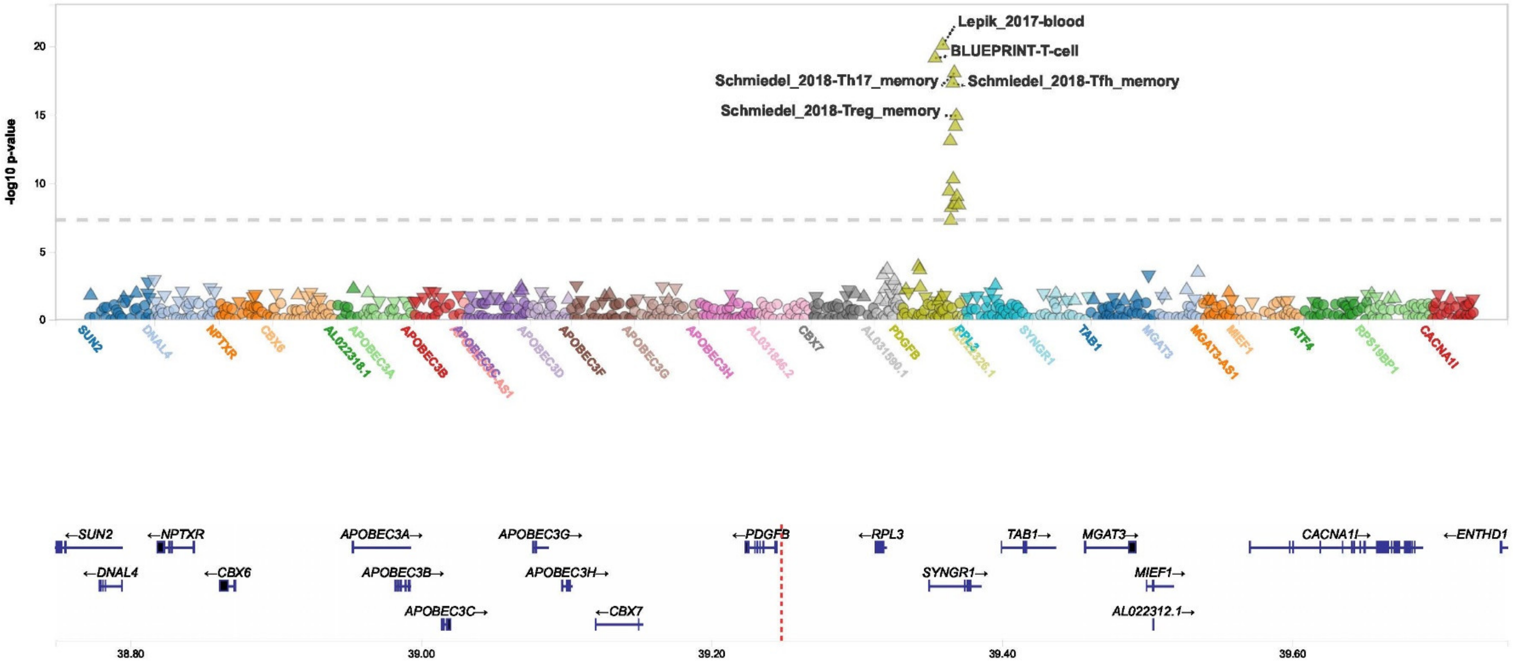

Lepik_2017-blood
BLUEPRINT-T-cell
Schmiedel_2018-Th17_memory
Schmiedel_2018-Tfh_memory
Schmiedel_2018-Treg_memory
-log10 p-value
0
5
10
15
20
SUN2
DNAL4
NPTXR
CBX6
APOBEC3A
APOBEC3B
APOBEC3D
APOBEC3F
APOBEC3G
APOBEC3H
CBX7
PDGFB
SYNGR1
TAB1
MGAT3
MGAT3-AS1
MIEF1
ATF4
CACNA1I
←SUN2
←NPTXR
←DNAL4
←CBX6
APOBEC3A→
APOBEC3B→
APOBEC3C→
APOBEC3G→
APOBEC3H→
←CBX7
←PDGFB
←RPL3
SYNGR1→
TAB1→
MGAT3→
MIEF1→
AL022312.1→
CACNA1I→
←ENTHD1
38.80
39.00
39.20
39.40
39.60

A.

B.

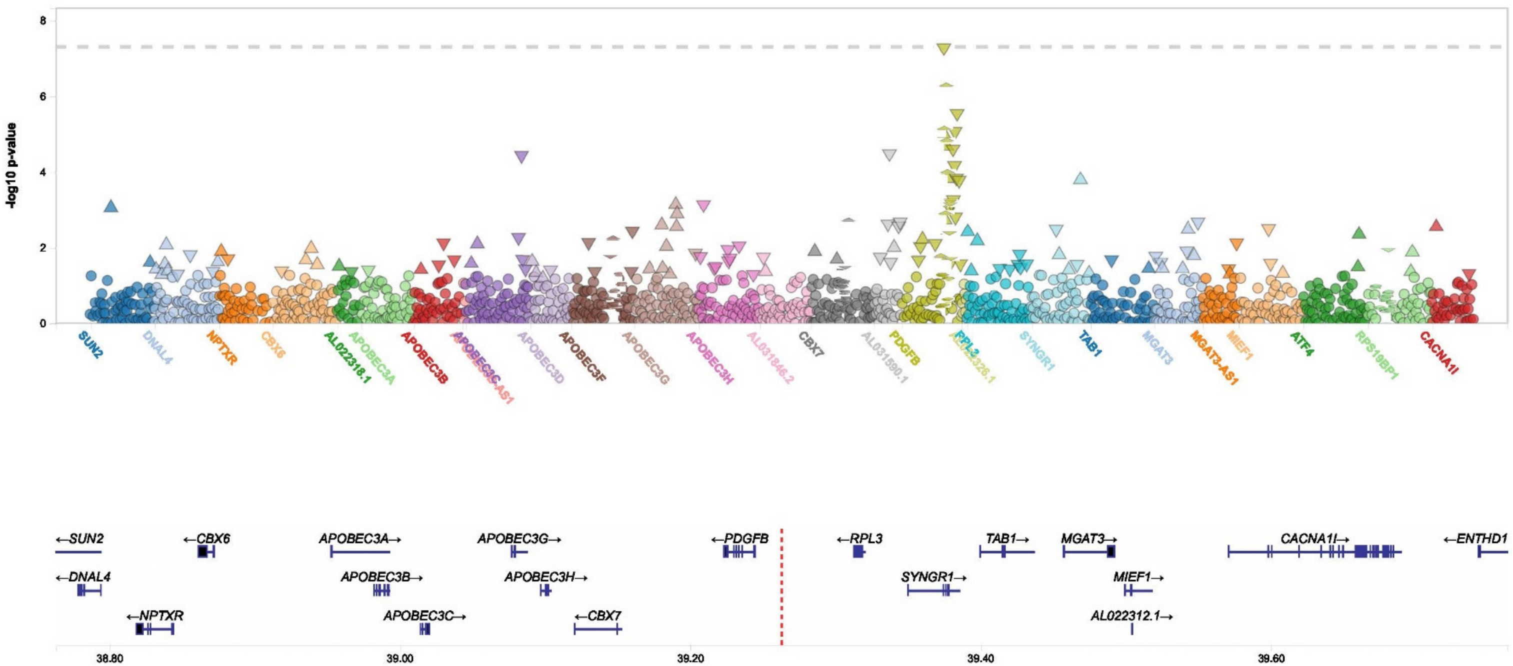

Figure S7. The eQTL P-values from the FIVEx database (https://fivex.sph.umich.edu) of **A.** rs130651 and **B.** rs4820371 with the genes located 500 kilobase pairs from the SNPs. These SNPs are significant cis-eQTLs for regulating *PDGFB* only. We exclude the non-coding genes. The red vertical dot lines indicate the locations of the SNPs. The top eQTL summary statistics are presented in **Table 2**.

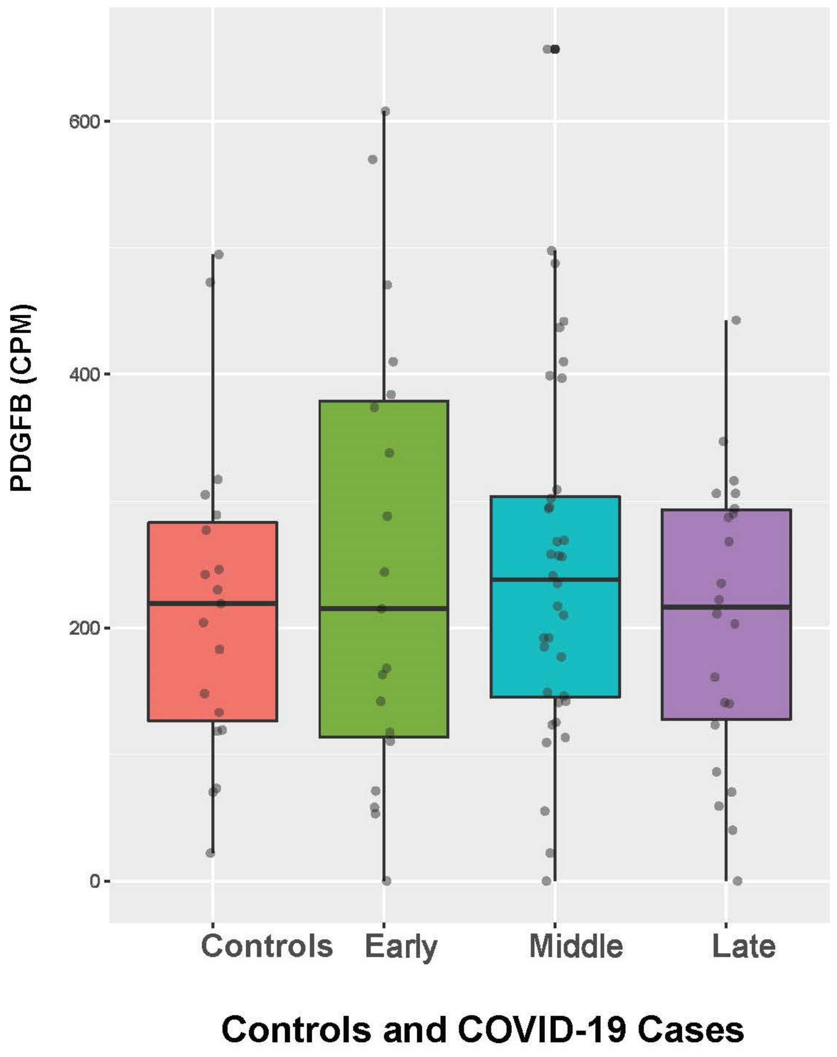

Figure S8. *PDGFB* gene expression levels across COVID-19-free controls and COVID-19 patients in the three different stages by the time after the symptom onset (early ≤10 days, middle 11-20 days, late ≥ 21 days). The Y-axis is the *PDGFB* gene expression level in the counts-per-million (CPM) unit.

A.

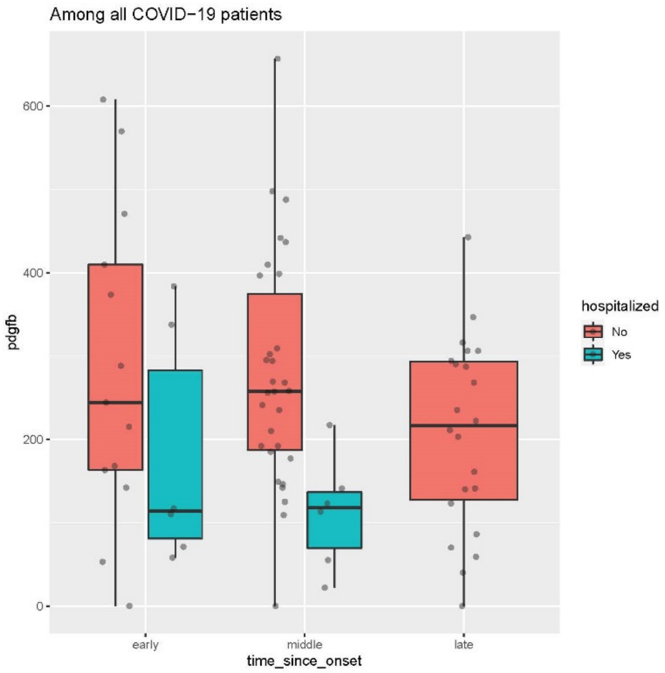

Among all COVID−19 patients
600
400
200
0
pdgfb
early
middle
late
time_since_onset
hospitalized
No
Yes

B.

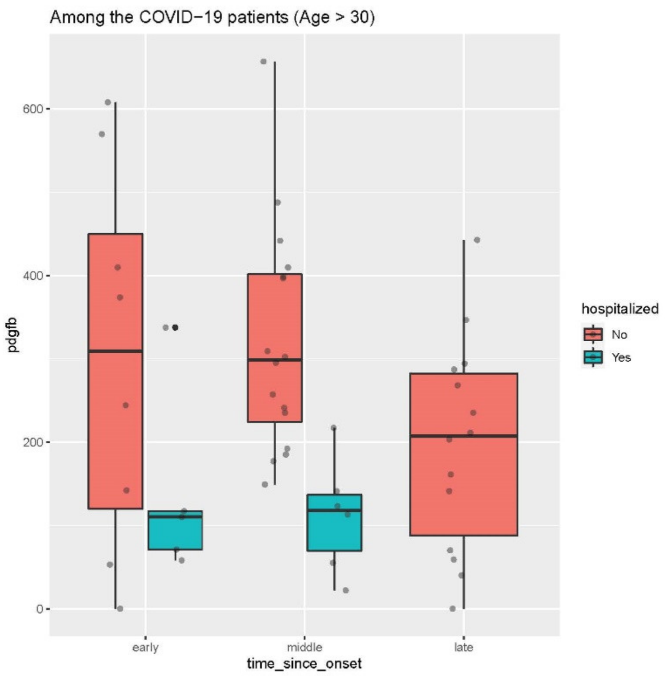
Among the COVID−19 patients (Age > 30)
600
400
200
0
pdgfb
early
middle
late
time_since_onset
hospitalized
No
Yes

C.

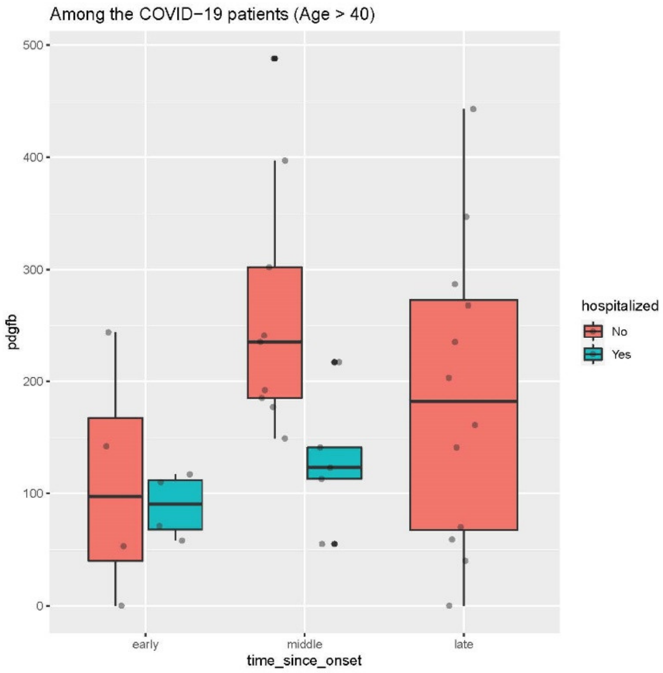

Among the COVID−19 patients (Age > 40)
500
400
300
200
100
0
pdgfb
early
middle
late
time_since_onset
hospitalized
No
Yes

D.

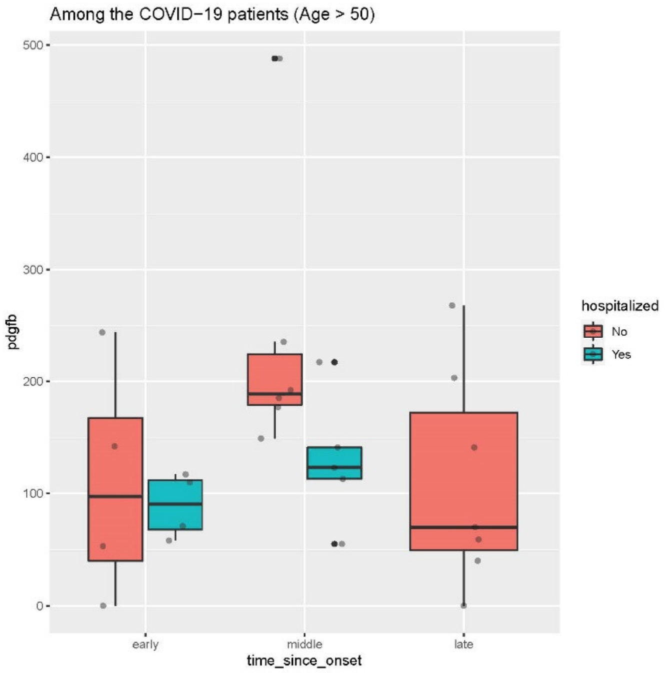

Figure S9. PDGFB gene expression levels at the three different stages of COVID-19 by the time after the symptom onset (early ≤10 days, middle 11-20 days, late ≥ 21 days) in different age groups: **A.** all sample, **B.** age > 30, **C.** age > 40, and **D.** age > 50.
